# Magnetic Extraction of Weathered Tire Wear Particles and Polyethylene Microplastics

**DOI:** 10.3390/polym14235189

**Published:** 2022-11-29

**Authors:** Vaibhav Budhiraja, Branka Mušič, Andrej Krzan

**Affiliations:** 1 Department of Polymer Chemistry and Technology, National Institute of Chemistry, Hajdrihova 19, 1000 Ljubljana, Slovenia; 2 Slovenian National Building and Civil Engineering Institute, Dimičeva ulica 12, 1000 Ljubljana, Slovenia

**Keywords:** degradation, magnetic separation, microplastics, polyethylene, tire wear particles

## Abstract

Magnetic extraction offers a rapid and low-cost solution to microplastic (MP) separation, in which we magnetize the hydrophobic surface of MPs to separate them from complex environmental matrices using magnets. We synthesized a hydrophobic Fe-silane based nanocomposite (Fe@SiO_2_/MDOS) to separate MPs from freshwater. Pristine and weathered, polyethylene (PE) and tire wear particles (TWP) of different sizes were used in the study. The weathering of MPs was performed in an accelerated weathering chamber according to ISO 4892-2:2013 standards that mimic natural weathering conditions. The chemical properties and morphology of the Fe@SiO_2_/MDOS, PE and TWP were confirmed by Fourier transform infrared spectroscopy and Scanning electron microscopy, respectively. The thermal properties of PE and TWP were evaluated by Thermogravimetric analysis. Using 1.00 mg of Fe@SiO_2_/MDOS nanocomposite, 2.00 mg of pristine and weathered PE were extracted from freshwater; whereas, using the same amount of the nanocomposite, 7.92 mg of pristine TWP and 6.87 mg of weathered TWP were extracted. The retrieval of weathered TWP was 13% less than that of pristine TWP, which can be attributed to the increasing hydrophilicity of weathered TWP. The results reveal that the effectiveness of the magnetic separation technique varies among different polymer types and their sizes; the weathering of MPs also influences the magnetic separation efficiency.

## 1. Introduction

Microplastics (MPs) are defined as plastic particles smaller than 5 mm [1]. They are found almost everywhere on Earth including oceans, wastewater, drinking water, sediments, soil, air, organisms and food [2,3]. Analysis of MPs’ presence involves a series of procedures starting with sample collection including environmental and organism samples, followed by treatments such as sieving, dissection, pre-digestion, etc. [4,5]. Afterwards, MPs are further separated using filtration, purification including enzymatic treatment, and are finally identified using analytical methods such as Fourier transform infra-red spectroscopy (FTIR), Raman spectroscopy, etc. [6]. The extraction of MPs from environmental samples is a challenging and crucial step for their effective analysis. The most common methods used for the separation of MPs are density separation, electrostatic separation, magnetic separation, oil and solvent separation [7,8]. Among these methods, density separation is most widely used as it is very effective although time-consuming, not suitable for collecting nano-plastics (NPs), high-density MPs such as TWP, paint particles, etc., and may discharge hazardous materials depending on the extraction solutions [9,10]. In particular, TWPs are difficult to separate and characterize from environmental matrices. It is therefore likely that a number of reports may have greatly underestimated the abundance of NPs and TWPs in the environment due to the challenges in identifying and isolating them [11]. A reliable analytical method to extract and characterize NPs and TWP is needed for effective analysis [12].

Magnetic separation is an emerging approach that is quick, low-cost and environmentally friendly. It is based on the principle of using hydrophobic adsorption to attach magnetic material to the hydrophobic surface of MPs. These can then be efficiently collected using a magnet, although the effectiveness of the method is strongly dependent upon the characteristics of the detected MPs including polymer type, properties, size and shape [7]. Using selective magnetization, we could overcome the shortcomings of current laboratory techniques for collecting NPs and TWP, as well as resolve environmental issues, such as wastewater treatment and those associated with environmental remediation. Iron-based nanoparticles found in the environment are byproducts of the weathering of iron-bearing minerals and biominerals, found in sediments, naturally occurring water sources, rocks and ash [13]. The fate, transport and chemical behavior of iron particles in environmental systems have already been studied and show no adverse effects on the environment [14].

Grbic et al., 2019 [15] demonstrated that small particles with a high surface to volume ratio are effectively separated by the magnetic separation method. They found that hydrophobic Fe nanoparticles can be used to bind plastic particles with a size below 20 µm. Misra et al., 2020 [16] have also reported an approach in which a polyoxometalate ionic liquid was adsorbed onto magnetic microporous core-shell Fe_2_O_3_/SiO_2_ particles that can remove MPs, organic, inorganic and microbial pollutants from water. Zhang et al., 2021 [17] have proved that a magnetic magnesium hydroxide coagulant made by incorporating magnetic Fe_2_O_3_ particles to polyacrylamide can be used to remove the PE MPs from wastewater. Tang et al., 2021 [18] have shown in their study that magnetic carbon nanotubes can be used to remove 100% of MPs from aqueous solutions. Furthermore, Kang et al., 2019 [19] have demonstrated that magnetic N-doped nanocarbon springs can be used to break down PE, polyvinyl chloride (PVC) and polypropylene (PP) into harmless substances in water. The advanced oxidation process provides a feasible strategy for MP pollution remediation in water. Recently, Shi et al., 2022 [20] have suggested the removal of PE MPs using a magnetic medium carrier (magnetic sepiolite) in a magnetic field created by a permanent magnet.

In the present work, the influence of the weathering of MPs on the magnetic separation was studied. A silane-coated Fe nanocomposite (Fe@SiO_2_/MDOS) was synthesized and used in the magnetic extraction of pristine and weathered PE and TWP from freshwater. A xenon light weathering chamber was used for artificial photodegradation for 1000 h according to the ISO 4892-2:2013 standard [21]. To the author’s knowledge, this is the first study in which weathered PE and TWP are extracted from freshwater using the magnetic separation method. We also evaluated the method’s suitability using road dust samples collected from the environment.

## 2. Materials and Methods

### 2.1. Chemicals and Materials

Iron nano powder, (99.5% metal basis, O < 10%), with a spherical morphology and an average particle size of 25 nm and a specific surface area of 40–60 m^2^/g was purchased from abcr (Karlsruhe, Germany). Ammonia solution 25% and absolute anhydrous ethanol were obtained from Merck (Darmstadt, Germany) and Carlo Erba (Val de Reuil, France) respectively. Tetraethyl orthosilicate (TEOS, 98%) was purchased from Sigma Aldrich (Steinheim, Germany) and methoxy(dimethyl)octadecylsilane (MDOS, 90%) was acquired from Sigma Aldrich (St. Louis, MO, USA). High Density Polyethylene (HDPE) (Polyethylene Lumicene Supertough 22ST05 and 32ST05) was procured from Total Energies (Felury, Belgium). Spherical polymethyl methacrylate (PMMA) nanoparticles with an average diameter of 670 nm were obtained from Evonik industries (Hanau, Germany). TWPs were taken from a Michelin passenger tire manufactured in April 2018 with the following type code: 245/45 R 18 100 V. Here, 245 is the section width, 45 is the aspect ratio, R stands for radial construction, 18 is the rim size and 100 and V represent load index and speed rating, respectively. The tread and sidewall of the tire had five and two plies, respectively. The tread plies had 2 polyester, 2 steel and 1 polyamide (PA) plies whereas both sidewall plies were made of polyester. Both PE and TWP were cryogenically ground using a ball mill Domel Tehtnica MillMix 20 (Domel, Zelezniki, Slovenia). Afterwards, the particles were sieved into different size ranges. For further experiments, PE was used in the size range of <100 µm and TWP in the size range of 300–600 µm. A permanent niodimium magnet (NdFeB N38) purchased from Svet Magnetov (Kamnik, Slovenia) was used for magnetic separation experiments. To analyze a real environmental sample, road dust was collected from the tunnel under the Ljubljana castle, Ljubljana, Slovenia (46°02′57.0″ N 14°30′37.4″ E) on 26 January 2022. The road dust in the size range of 63–125 μm was sieved and used during the experiment.

### 2.2. Accelerated Ageing

A xenon light weathering Q-SUN Xe-3 chamber (Q-Lab, Bolton, UK, Europe) was used for artificial photodegradation lasting up to 1000 h. The chamber was operated according to the ISO 4892-2:2013 standard [21]. This chamber enables the setting up of different accelerated weathering conditions by changing the UV irradiation power, relative humidity and temperature to perform the laboratory-accelerated aging of plastic materials. The relative humidity in the chamber was regulated by an ultrasound humidifying system. Its three air-cooled 1800 W xenon lamps emit light in the 300–400 nm wavelength range. At a fan speed of 2000 rpm, the substrates were subjected to 60 W/m^2^ of irradiation at 38 °C in the chamber, 65 °C for the black standard and 50% relative humidity [21,22]. After 1000 h, samples were taken out of the weathering chamber and stored in a dark envelope until further experiments.

### 2.3. Synthesis of Fe@SiO_2_/MDOS Nanocomposite

A magnetic iron silane nanocomposite (Fe@SiO_2_/MDOS) was synthesized by a two-step process. In the first step, Fe nanoparticles were coated with silica using the sol-gel process: Fe nanoparticles (1.00 g) were dispersed in a solvent mixture of ethanol (160 mL) and water (40 mL) using an ultrasonic bath. TEOS (3 mL) and ammonia (4 mL) were added to this suspension, which was then continuously stirred for 8 h. During the stirring, TEOS would hydrolyze and create the silica oligomers around the Fe nanoparticles. The Fe@SiO_2_ nanocomposites collected by centrifugal separation were washed with ethanol and Milli-Q water. The washed Fe@SiO_2_ nanocomposites were dried overnight in a vacuum chamber at 50 °C. 

In the second step, MDOS was then grafted onto the surface of the Fe@SiO_2_ nanocomposites using the silane chemistry of MDOS: Typically, 50 mL of anhydrous ethanol was used to homogeneously combine 1.00 g of Fe@SiO_2_ nanocomposite and 0.50 g of MDOS before being further sonicated for 20 min. The final mixture was swirled for 8 h at reflux temperature under a nitrogen atmosphere. The MDOS-modified Fe@SiO_2_ nanocomposite, Fe@SiO_2_/MDOS was cooled naturally to room temperature, centrifuged with anhydrous ethanol to remove excess organo-silane and then dried under reduced pressure at 50 °C [23,24].

### 2.4. Characterization of Fe@SiO_2_/MDOS, PE and TWP

The Fe@SiO_2_/MDOS nanocomposite, PE and TWP particles were characterized by Attenuated Total Reflectance-Fourier Transform Infrared spectroscopy (ATR-FTIR) using a Perkin-Elmer Spectrum One (Perkin Elmer, Waltham, MA, USA) Fourier transform infra-red spectrometer combined with an attenuated total reflectance diamond crystal attachment measuring in the 4000–600 cm^−1^ range. Substrates were evaluated before and after exposure to accelerated aging. The resulting ATR-FTIR spectra were validated by direct visual inspection with the ATR-FTIR spectra collection and compared to the spectra from the reference spectral library, IR Hummel Industrial Polymers Volume 3 (c) 1999 Chemical Concepts GmbH. 

Thermogravimetric analysis (TGA) of PE, weathered PE_1000h_, TWP and weathered TWP_1000h_ were carried out on a Mettler Toledo (Greifensee, Switzerland) TGA/DSC 1 thermogravimeter. For PE, the experiments were carried out in the temperature range of 40–650 °C. The experiments were performed in a nitrogen atmosphere with a flow rate of 50 mL/min and a heating rate of 30 K/min. For TWP, the experiments were carried out in the temperature range of 30–900 °C. The experiments were done in an N_2_ environment with a flow rate of 30 mL/min and a heating rate of 20 K/min from 30–700 °C and in an O_2_ atmosphere with a flow rate of 30 mL/min and a heating rate of 20 K/min from 700–900 °C. 

The surface morphology of Fe@SiO_2_/MDOS nanocomposite, PE, weathered PE_1000h_, TWP and weathered TWP_1000h_ were examined on a JEOL model JSM-IT500LV, Oxford Instruments (Tokyo, Japan) scanning electron microscope equipped with an EDS analyzer suitable for analyzing the elemental composition, with the following technical specifications: high vacuum mode, resolution of 3.00 nm (30 kV) and 15.00 nm (1.00 kV) in high vacuum mode, resolution of 4.00 nm (30 kV, BED), electron gun: W filament, fully automatic gun alignment, accelerating voltage from 0.3 to 30 kV, probe current from 1 pA to 1 µA, maximum specimen size: 200 mm (diameter) × 75 mm (height). To avoid electrostatic charge accumulation during observation, gold was sputter-coated onto each sample. At 10 kV, images at various magnifications were captured. 

### 2.5. Recovery of PE and TWP

PE particles and TWP were magnetically extracted from freshwater using a NdFeB N38 magnet. The magnet was attached to a stainless steel rod, which was dipped and swirled in the sample to collect MPs. Afterward, the rod was removed and rinsed with deionized water to collect MPs in the petri dish. Using this method, the TWP were easily separated, dried and weighed. The PE MPs were recovered by putting the rod into the ultrasonic bath for 30 sec; the MPs then detach from the magnet and afterward they are sieved and collected to be weighed. Each experiment was repeated three times and standard deviations in the results are reported.

## 3. Results and Discussion

### 3.1. Attenuated Total Reflectance-Fourier Transform Infrared Spectroscopy

**Figure 1:**
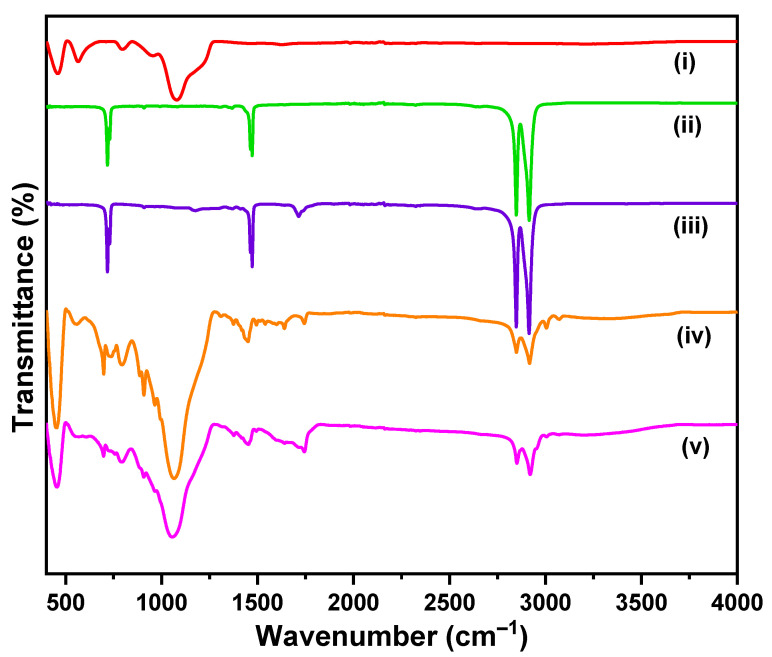
FTIR spectra of (i) Fe@SiO_2_/MDOS (ii) PE (iii) weathered PE_1000h_ (iv) TWP and (v) weathered TWP_1000h_.

The FTIR spectra depicted in Figure 1 show that (i) Fe@SiO_2_/MDOS nanocomposite exhibits a strong band at 1076 cm^–1^ that corresponds to Si-O asymmetric stretching vibration. The bands at 454 cm^−1^ and 791 cm^−1^ are assigned to Si-O symmetric stretching and bending vibration, respectively [25]. The significant band at 564 cm^−1^ is attributed to Fe-O bending vibration, confirming the formation of iron-silane nanocomposite [26]. The spectra of (ii) PE and (iii) weathered PE_1000h_ exhibit specific PE peaks independent of weathering. The strong bands at 2915 cm^−1^ and 2848 cm^−1^ are assigned to the CH_2_ asymmetric stretching and CH_2_ symmetric stretching respectively. The band at 1469 cm^−1^ is credited to the crystallinity effects of CH_2_ bending deformation, whereas the band around 730 cm^−1^ arises from a skeletal vibration of (-CH_2_-) in the polymer chain [27]. A strong IR absorbance band between 1710 cm^−1^ and 1740 cm^−1^ is observed for weathered PE_1000h_ that corresponds to the carbonyl group (C=O), confirming the oxidation of the polymer [21,28]. The spectra of (iv) TWP and (v) weathered TWP_1000h_ exhibit similar peaks independent of weathering. It is difficult to assign peaks specific to TWP because of its complex chemical composition, which contains carbon black in the rubber mixture. However, an IR absorbance band between 1710 cm^−1^ and 1740 cm^−1^ is observed for weathered TWP_1000h_ that corresponds to a carbonyl group (C=O), implying surface oxidation.

The carbonyl Index (CI) is used to evaluate the oxidation and characterize the degree of aging of PE and TWP. The CI is calculated using the “specified area under band” (SAUB) method [29]. The CI is defined as the ratio of area under band 1850–1650 cm^−1^ and area under 1500–1420 cm^−1^ which correspond to carbonyl (C=O) and methylene (CH_2_) peaks, respectively [29]. The values of CI for PE, weathered PE_1000h_, TWP and weathered TWP_1000h_ were 0.279, 0.701, 0.798 and 2.551, respectively. The values of CI are directly proportional to the surface oxidation of PE and TWP. These results are supported by Scanning Electron Microscopy (SEM) micrographs.

### 3.2. Thermogravimetric Analysis

**Figure 2:**
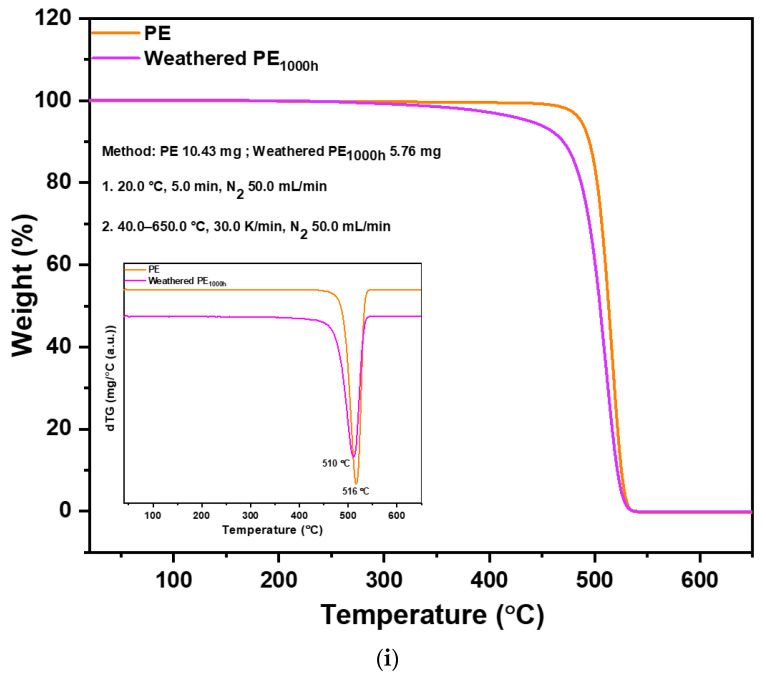

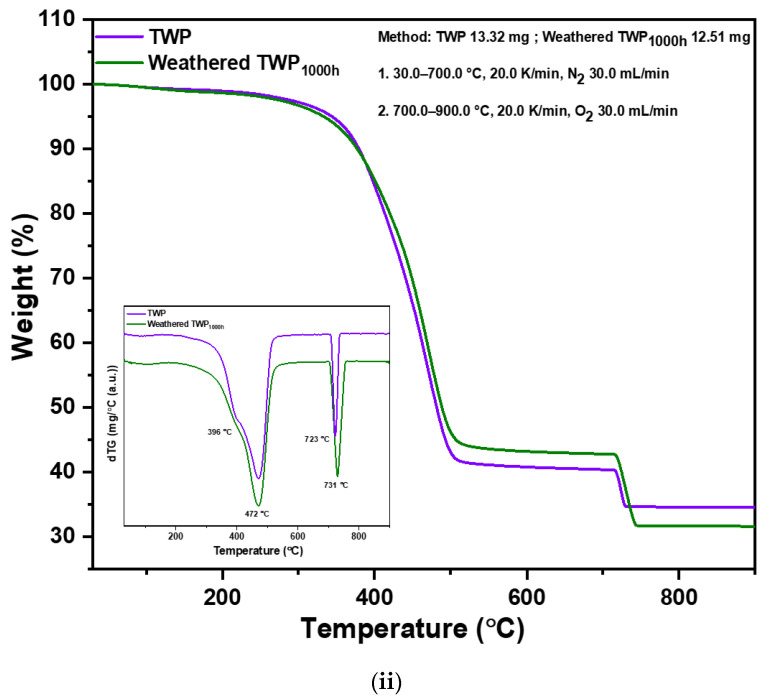
TGA of (**i**) PE, weathered PE_1000h_ (**ii**) TWP, weathered TWP_1000h_. First differentials are shown in the inset.

The TGA confirmed the difference in thermal properties between pristine and weathered PE and TWP. The TGA of PE, weathered PE_1000h_, TWP and weathered TWP_1000h_ are depicted in Figure 2i,ii, respectively. The weathered PE_1000h_ and TWP_1000h_ showed a decrease in thermal stability as compared to pristine PE and TWP, respectively, as seen by the decrease in onset temperature and temperature of the maximum mass loss rate. Table 1 depicts the onset temperature (T_os_), midset temperature (T_mid_, T_50%_), maximum degradation temperature (T_max_) and first derivative peak temperature, also called the inflection point (T_p_). The results show a significant reduction in the thermal stability of weathered PE MPs compared to pristine PE, which is confirmed by a decrease in the T_os_ to 467 °C for weathered PE as compared to the T_os_ value of 486 °C for pristine PE. The T_mid_ and T_max_ values for PE and weathered PE show comparable figures. The T_mid_ is an important indicator for the thermal stability of the polymer. The overall degradation in weight remains similar for pristine and weathered PE. This leads to the conclusion that the partially weathered PE becomes more reactive as compared to the pristine PE [21,30].

Tires from different means of transport and from different manufacturers differ in their chemical composition. An automobile tire may contain more than 100 components such as natural rubber, synthetic rubber, steel, nylon, silica (derived from sand), polyester, carbon black, petroleum and many more additives present in lower concentrations. Generally, a tire consists of softeners: oils, resins (15 mass%), polymers: natural rubber, synthetic rubber (40–50 mass%), fillers: carbon black, silica, chalk (30–35 mass%), vulcanization agents: sulfur, zinc (2–5 mass%) and various additives (5–10 mass%) [31]. Due to the complex chemical composition of tires, it is expected that mass losses from the various compounds contained in the rubber will overlap, especially in the temperature range, where the overall mass loss is greatest, between 200 and 500 °C. As shown in Figure 2ii, pristine TWP has a shoulder at 396 °C which is not present in the weathered TWP. During the experiment, we found that a layer of oil was formed on the quartz glass due to oil evaporation. This explains the disappearance of a peak in weathered TWP and led to the conclusion that TWP can cause uncontrollable oil release into the environment. The weight loss at 472 °C is due to the thermal degradation of the polymer. Due to the slow release of oils from the tire, it overlaps with the weight loss curve of the weathered polymer. Therefore, it is difficult to accurately measure the mass of oil and polymer in the tire. In the sample, an endothermic process occurred at temperatures of 723 °C and 731 °C, respectively in which energy was used for the process, which also manifests itself in mass loss. Due to the high temperature where the process takes place, the mass loss due to carbon black occurs in this temperature range; the decomposition curve at 723 °C and 731 °C is attributed to the weight loss of carbon black [32]. The values of T_os_, T_mid_, T_max_ and T_p_ for TWP and weathered TWP_1000h_ are represented in Table 1.

### 3.3. Scanning Electron Microscopy

**Figure 3:**
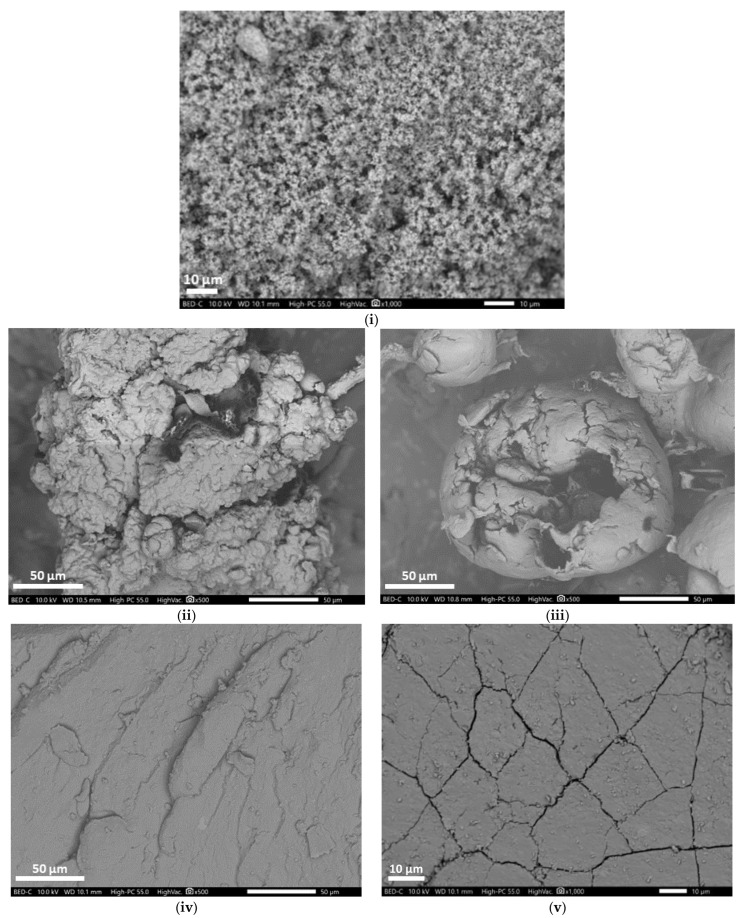
SEM of (**i**) Fe@SiO_2_/MDOS (**ii**) PE (**iii**) Weathered PE_1000h_ (**iv**) TWP (**v**) Weathered TWP_1000h_.

A representative SEM image of Fe@SiO_2_/MDOS is shown in Figure 3i. The image was taken at 10 kV with 1000 magnification. It shows small globular granules, confirming that a hydrophobic silane coating layer was formed around the surface of iron particles [33]. Figure 3ii,iii show the surface morphology of PE and weathered PE_1000h,_ respectively. The image was taken at 10 kV with 500 magnification. The PE surface is uneven and rough with some cracks, whereas the weathered PE_1000h_ has more pronounced cracks with pores developing on the surface due to oxidation, as confirmed by FTIR studies [34]. Figure 3iv,v depict the surface morphology of TWP and weathered TWP_1000h_ taken at 10 kV with 500 and 1000 magnification, respectively. The TWP has a layered surface with some bumps over the surface, whereas weathered TWP_1000h_ has a fractured surface due to oxidation of the TWP, as revealed in CI values calculated by the SAUB method [35]. 

It is clear from the SEM images that UV radiation causes changes on the surface of polymer materials due to oxidative aging of the TWP and PE MPs. As a result of the accelerated aging, polymer materials decompose, eventually breaking down into NPs that accumulate in nature. The problem is exacerbated by the fact that 10^9^ tires [36] and over 360 metric tons of fossil-based polymers are produced worldwide annually, with an 8.4% annual increase rate [37]. These polymers, after their intended use, find their way into the environment as plastic litter.

### 3.4. Magnetic Extraction Experiments

**Figure 4:**
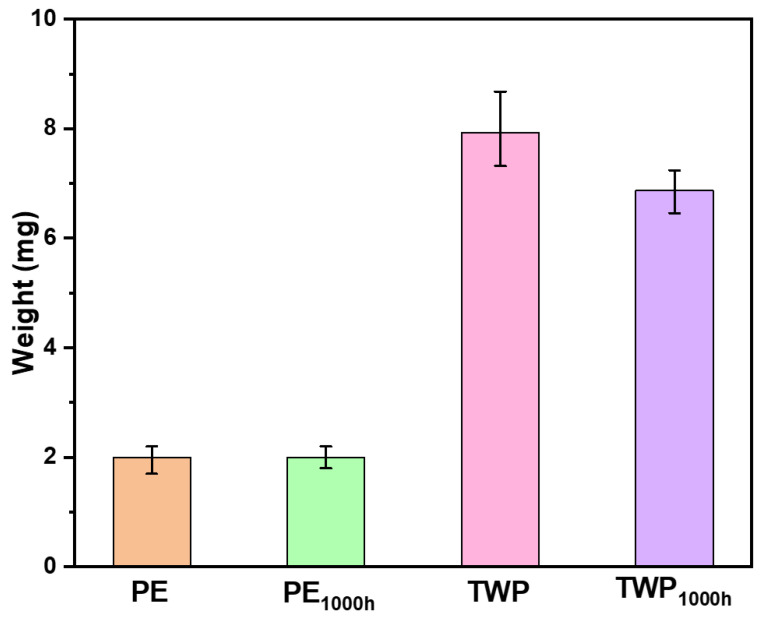
Results from recovery experiments of PE, Weathered PE_1000h_ (<100 µm) and TWP, Weathered TWP_1000h_ (300–600 µm). Error bars show standard deviation.

The magnetic extraction of pristine and weathered PE and TWP has been studied. For each 1.00 mg of Fe@SiO_2_/MDOS nanocomposite, approximately 2.00 ± 0.25 mg of pristine and 2.00 ± 0.20 mg of weathered PE have been extracted using a permanent NdFeB magnet in freshwater. Similarly, for 1.00 mg of Fe@SiO_2_/MDOS nanocomposite, 7.92 ± 0.68 mg and 6.87 ± 0.39 mg of pristine and weathered TWP have been extracted. The experiments were repeated three times and the standard deviation is reported as error bars depicted in Figure 4. The large difference in the mass of PE and TWP being retrieved is due to their sizes. The smaller the particle size, the greater is its surface area, therefore 2.00 mg of pristine PE (<100 µm) and 7.92 mg of pristine TWP (300–600 µm) were extracted. Weathered TWP retrieval was 13% lower than that of pristine TWP, which can be attributed to weathered TWP’s increasing hydrophilicity. However, weathered PE MPs do not show any significant effect on the magnetic separation technique; this could be because the extent of degradation does not substantially affect the surface properties. The CI for PE and weathered PE_1000h_ was found to be 0.279 and 0.701, respectively, whereas for TWP and weathered TWP_1000h_ the value increases from 0.798 to 2.551, respectively. The high CI value for weathered TWP_1000h_ implies that the TWP is highly oxidized and has a hydrophilic surface. While the weathered PE_1000h_ is oxidized, the extent of oxidation is still low compared to the weathered TWP_1000h_. Shi et al., 2022 [38] have shown in their study that the removal of PE, PP, Polystyrene (PS) and Polyethylene Terephthalate (PET) MPs in the size range of 200–900 μm varies due to differences in their physiochemical properties such as density, crystallinity etc. The differential scanning calorimetry of pristine and weathered PE MPs has been carried out to confirm the effect of crystallinity on magnetic extraction of PE. The results revealed that the crystallinity of PE is 74%, which was reduced to 68% after the degradation. The degradation does not affect the crystallinity to a great extent; this could also be a reason for no change in the extraction of pristine and weathered PE MPs. The DSC of PE and PE_1000h_ is shown in Supplementary Figure S1. 

The results have shown that the magnetic extraction capacity of weathered MPs with Fe@SiO_2_/MDOS nanocomposite decreases compared to the pristine MPs for two reasons. Firstly, the weathered MPs have a more hydrophilic surface due to surface oxidation [39,40]. Secondly, the weathered MPs can fragment during the experiment, which leads to an increase in the surface area of the MPs and thus affects their overall extraction capacity [15,41]. So, the hydrophilicity and surface area of MPs can affect the magnetic extraction of MPs. All previous studies demonstrating the magnetic extraction of MPs from different environmental samples have utilized pristine MPs in their experiments [15]. These results confirm that the behaviour of weathered MPs is different from pristine MPs, which should be relevant to the study of real-world MP pollution, since in the environment we encounter degraded MPs whose properties vary significantly from those of pristine MPs [42]. 

Furthermore, PMMA NPs and a road dust sample collected from the environment were analyzed to verify the applicability of the method to real-life samples. Even though PMMA NPs were successfully separated from the freshwater, counting their number could not be performed due to their size and continuous motion. It was impossible to keep the NPs within a microscope’s field of view. Recently, Martin et al., 2022 [43] have shown that 90% of the NPs in the size range of 100–1000 nm were magnetically separated from water using iron oxide nanoparticles (IONP). They used fluorescently labelled PS to show the quantification of PS NPs interacting with IONP using SEM imaging and fluorescence. 

The majority of the road dust (60%) is made up of road-derived minerals, with quartz making up the majority (40–50%) and the remaining clay-forming minerals coming from nearby soils. Organic matter, mostly composed of plant matter, made up around 2% of the total. About 30% of the accumulation was made up of potentially hazardous contaminants including road wear particles, fly ash from asphalt, TWP, heavy metals and particulate combustion emissions [44]. Because there were fewer particle sources and no precipitation, road dust collected in tunnels would contain more TWP than dust collected from open highways; however, due to the lack of sunlight and rain, TWP in tunnel dust is less exposed to sunlight and rain [45]. Road dust collected from the tunnel was sieved to a size range of 63–125 μm. The TGA of this fraction showed a small peak at 443 °C which could be due to the degradation of the polymer in tire rubber. The TGA of road dust is shown in Supplementary Figure S2. The weathered TWP_1000h_ were added to the road dust sample collected from the tunnel in the ratio of 1:2 by weight. The recovery rate of TWP from road dust samples was found between 59–77%. The road dust sample collected from the environment also contains TWP that is not considered, as only the weathered TWP_1000h_ added was measured. The magnetic extraction of PE and TWP from freshwater is shown in Supplementary Videos S1 and S2, respectively. The magnetic separation of several MPs in different size ranges separated by various materials is discussed in detail in Table 2.

## 4. Conclusions and Future Perspectives

This work provides a feasible strategy for the magnetic extraction of pristine and weathered PE and TWP from freshwater using an iron-silane based Fe@SiO_2_/MDOS nanocomposite. The formation of Fe@SiO_2_/MDOS and the oxidation of weathered PE and TWP are assessed by spectral change in ATR-FTIR spectroscopy. The change in thermal properties and morphology of PE and TWP is confirmed by TGA and SEM analysis. The large difference in the recovery of PE and TWP is mainly due to their different sizes. The retrieval of weathered TWP was 13% lower than that of pristine TWP, which can be attributed to the increasing hydrophilicity of weathered TWP. Herein, we have demonstrated that binding and recovery of pristine and weathered PE and TWP depend upon the surface area and hydrophobic/hydrophilic surface of the MPs. Therefore, it is important to use weathered MPs for magnetic extraction experiments, which gives us more reliable results as it is based on the surface interaction of material with the MPs.

The magnetic separation technique has the capability of removing NPs from the environment, which is simple, economical and less time-consuming. Iron-based nanocomposites are a viable candidate for use in water remediation and the removal of MPs/NPs, as they are economical to produce and environmentally friendly. However, further research and development is needed to make iron-based environmentally friendly nanocomposites with better magnetic properties that can remove various contaminants including dyes, heavy metals, MPs, NPs, etc., for improved environmental remediation. Further advancements in magnetic separation toward large-scale applications could be made possible by the construction of novel, inexpensive magnetic carrier media and separation facilities. In summary, this work demonstrates the viability of a rapid, economical and effective magnetic extraction method to separate MPs from freshwater. The results presented here may serve as a solid foundation for future research that should be concerned with the extraction of NPs and the scaling up of this process with environmental samples.

## Figures and Tables

**Table 1:** Thermal profile determined for (i) PE (ii) weathered PE_1000h_ (iii) TWP (iv) weathered TWP_1000h_.

Polymer	T_os_ (°C)	T_mid_ (°C)	T_max_ (°C)	T_p_ (°C)
PE	485.96	513.25	525.17	515.92
Weathered PE_1000h_	466.97	505.64	521.73	510.22
TWP	342.41	481.46	727.62	472.00 723.00
Weathered TWP_1000h_	333.09	488.40	740.51	472.00 731.00

**Table 2:** Reported literature on the magnetic separation of the MPs.

Material	MPs Type	MPs Size	Removal Efficiency	Reference
Fe-Hexadecyltrimethoxysilane	HDPE, PP, PS, PU ^1^, PVC, PET	200–1000 µm	49–105%	[15]
Magnetic polyoxometalate ionic-liquids	PS	1 or 10 µm	100%	[16]
Magnetic Carbon Nanotubes	PE, PA, PET	48 µm	100%	[18]
Magnetic Sepiolite	PE	48 µm	98.4%	[20]
Nano-Fe_3_O_4_	PE, PP, PS, PET	200–900 µm	62.83–86.87%	[38]
Fe-polydimethylsiloxane	LDPE ^2^ PS	2–5 mm 100–1000 nm	100% 90%	[43]
Magnetic biochar	PS	1 µm	94.8%	[46]
Magnetic seeded filtration, Fe_3_O_4_	PVC, PMMA	2.06–5.98 µm	95%	[47]
Magnetic steel collector wire	PE, PET, PTFE ^3^	63 µm–2 mm	87–97%	[48]
Carbon/iron nanocomposite	PS	147 µm	100%	[49]
Fe_3_O_4_	PE, PP, PVC, PS, PET	20–800 µm	100%	[50]
Magnetic seeded filtration, Fe_3_O_4_	LDPE, PP, PVC, PS, PET	100 µm	80–100%	[51]
Fe_3_O_4_ polyoxometalate/n-octylamine	PS, PET, Polysulphone	1–500 µm	83–99%	[52]
Fe_2_O_3_-MnO_2_ micromotor	PE	0.8 µm	10%	[53]
Fe_3_O_4_	PS, PMMA	105–970 nm	-	[54]
Fe-Lauric Acid	HDPE	273–1250 µm	100%	[55]
Polydimethylsiloxane-Ni Foam	LDPE, PP, PS, PC, PET, PVC, PTFE	70–1200 µm	86.25–100%	[56]
Magnetic microsubmarines, sunflower pollon grains	PS	40–100 µm	70–75%	[57]
Fe@SiO_2_/MDOS	PE TWP	<100 µm 300–600 µm	- 59–77%	Present Work

^1^ Polyurethane, ^2^ Low Density Polyethylene; ^3^ Polytetrafluoroethylene.

## Data Availability

The data presented in this study are available on request from the corresponding author.

## Supplementary Materials

The following supporting information can be downloaded at: https://www.mdpi.com/article/10.3390/polym14235189/s1, Figure S1: DSC of PE and weathered PE_1000h_; Figure S2: TGA of Road Dust. First differential is shown in the inset; Video S1: Magnetic Extraction of Polyethylene; Video S2: Magnetic Extraction of Tire Wear Particles.
