# Supplementary material for: Magnetic Extraction of Weathered Tire Wear Particles and Polyethylene Microplastics
Source: Polymers (Basel). 2022 Nov 29;14(23):5189. doi: 10.3390/polym14235189 (PMC9740573; doi:10.3390/polym14235189)
Supplement: Supplementary file 1 [file polymers-14-05189-s001.zip › Supplementary.pdf]

Supplementary Material to:

Magnetic Extraction of Weathered Tire Wear Particles and Polyethylene Microplastics

Vaibhav Budhiraja [1,*], Branka Mušič [2,*] and Andrej Krzan [1]

1. Department of Polymer Chemistry and Technology, National Institute of Chemistry, Hajdrihova 19, 1000 Ljubljana, Slovenia
2. Slovenian National Building and Civil Engineering Institute, Dimičeva ulica 12, 1000 Ljubljana, Slovenia

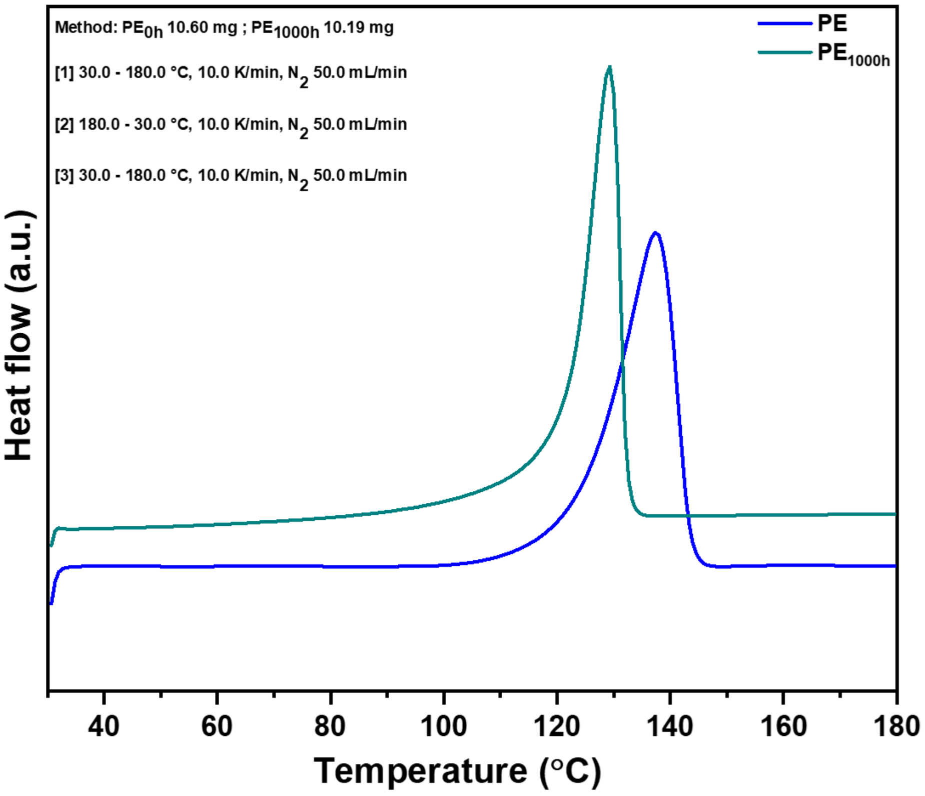

Figure S1. DSC of PE and weathered PE_{1000h}

The DSC of PE and weathered PE_{1000h} has been done with a Mettler Toledo, Greifensee, Switzerland TGA/DSC 1 thermogravimeter. The experiments were done in the temperature range of 30-180 °C in an environment of N_2, with a flow rate of 50 mL/min and a heating rate of 10 K/min. The crystallinity of the PE is calculated using the formula;

$$\text{Degree of Crystallinity (\%)} = \frac{\Delta H_m J/g \text{ (calculated)}}{\Delta H_m J/g \text{ (100\%)}} \times 100$$

The melting enthalpy of a 100% crystalline PE used for calculation is 293 J/g. The crystallinity of pristine PE and weathered PE_{1000h} is 74% and 68% respectively.

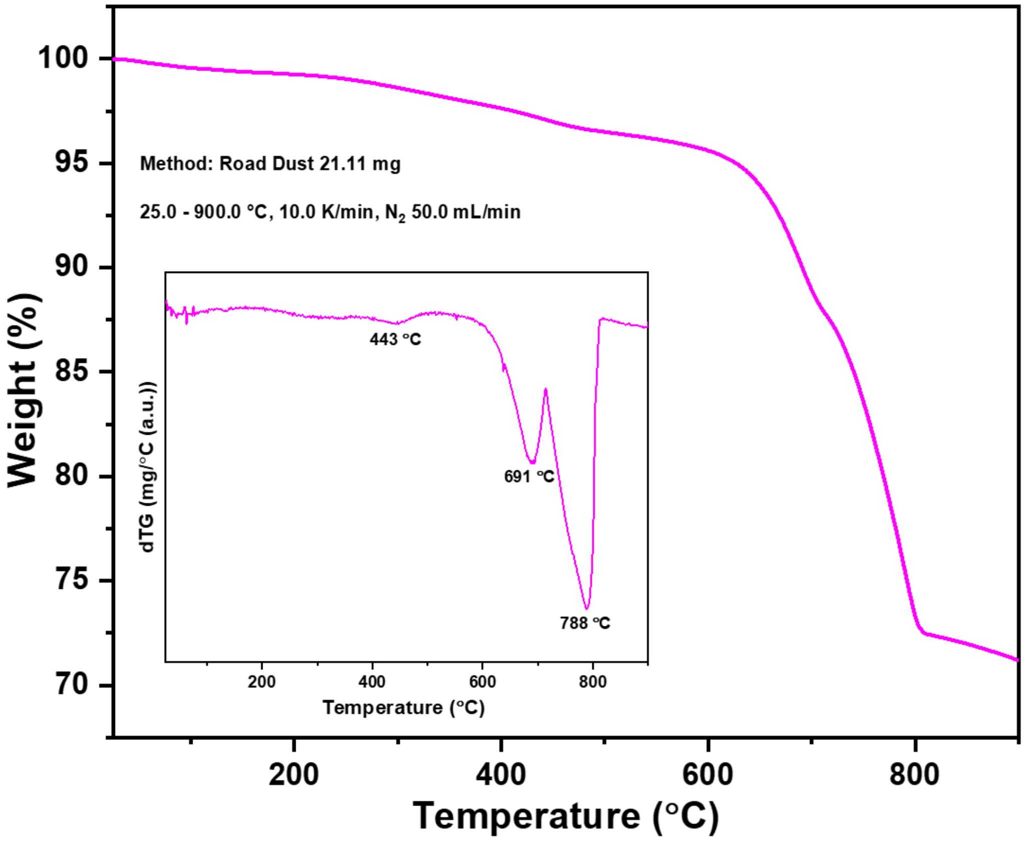

Figure S2. TGA of Road Dust. First differential is shown in the inset.

The TGA of road dust has been done with a Mettler Toledo, Greifensee, Switzerland TGA/DSC 1 thermogravimeter. The experiments were done in the temperature range of 25-900 °C in an environment of N_2, with a flow rate of 50 mL/min and a heating rate of 10 K/min. The first derivative shows a mild peak at 443 °C which could be from the degradation of tire rubber. The peak at 691 °C could be from carbon black originating from the tire or any other source in the dust sample. The most prominent peak at 788 °C might be due to some inorganic impurities in the dust sample.
